# Stromal Notch ligands foster lymphopenia-driven functional plasticity and homeostatic proliferation of naive B cells

**DOI:** 10.1172/JCI158885

**Published:** 2022-07-01

**Authors:** Daniela Gómez Atria, Brian T. Gaudette, Jennifer Londregan, Samantha Kelly, Eric Perkey, Anneka Allman, Bhaskar Srivastava, Ute Koch, Freddy Radtke, Burkhard Ludewig, Christian W. Siebel, Russell J.H. Ryan, Tanner F. Robertson, Janis K. Burkhardt, Warren S. Pear, David Allman, Ivan Maillard

**Affiliations:** 1 Division of Hematology/Oncology, Department of Medicine,; 2 Department of Pathology and Laboratory Medicine, Perelman School of Medicine, and; 3 Immunology Graduate Group, University of Pennsylvania, Philadelphia, Pennsylvania, USA.; 4 Graduate Program in Cellular and Molecular Biology, University of Michigan, Ann Arbor, Michigan, USA.; 5 EPFL, Lausanne, Switzerland.; 6 Medical Research Center, Kantonsspital St. Gallen, St. Gallen, Switzerland.; 7 Department of Discovery Oncology, Genentech, South San Francisco, California, USA.; 8 Department of Pathology, University of Michigan, Ann Arbor, Michigan, USA.; 9 Children’s Hospital of Philadelphia, Philadelphia, Pennsylvania, USA.

**Keywords:** Immunology, Adaptive immunity, Immunoglobulins

## Abstract

In lymphopenic environments, secondary lymphoid organs regulate the size of B and T cell compartments by supporting the homeostatic proliferation of mature lymphocytes. The molecular mechanisms underlying these responses and their functional consequences remain incompletely understood. To evaluate homeostasis of the mature B cell pool during lymphopenia, we turned to an adoptive transfer model of purified follicular B cells into *Rag2^–/–^* mouse recipients. Highly purified follicular B cells transdifferentiated into marginal zone–like B cells when transferred into *Rag2^–/–^* lymphopenic hosts but not into wild-type hosts. In lymphopenic spleens, transferred B cells gradually lost their follicular phenotype and acquired characteristics of marginal zone B cells, as judged by cell surface phenotype, expression of integrins and chemokine receptors, positioning close to the marginal sinus, and an ability to rapidly generate functional plasma cells. Initiation of follicular to marginal zone B cell transdifferentiation preceded proliferation. Furthermore, the transdifferentiation process was dependent on Notch2 receptors in B cells and expression of Delta-like 1 Notch ligands by splenic Ccl19-Cre^+^ fibroblastic stromal cells. Gene expression analysis showed rapid induction of Notch-regulated transcripts followed by upregulated *Myc* expression and acquisition of broad transcriptional features of marginal zone B cells. Thus, naive mature B cells are endowed with plastic transdifferentiation potential in response to increased stromal Notch ligand availability during lymphopenia.

## Graphical abstract

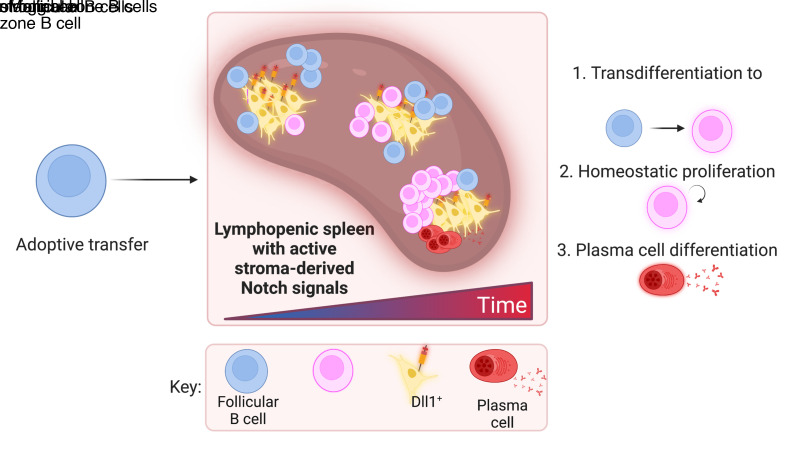

## Introduction

Multiple factors control the size and composition of lymphocyte pools in secondary lymphoid organs. Both de novo production and attrition of mature lymphocytes through cell death or differentiation are critical determinants of lymphocyte counts in health and disease. In addition, naive mature lymphocytes can undergo homeostatic proliferation in response to lymphopenic environments, an important mechanism to sustain lymphocyte numbers when production is insufficient and/or attrition is accelerated. Although lymphopenia is defined clinically based on a low absolute lymphocyte count in the blood, these numbers typically reflect changes in lymphocyte counts within secondary lymphoid organs. In patients, prolonged lymphopenia is associated with several prevalent contexts, including aging, viral infections, autoimmune disorders, bone marrow transplantation, and the administration of myeloablative or lymphodepleting anticancer therapy. Yet, the molecular mechanisms underlying homeostatic responses to lymphopenia and their functional consequences remain incompletely understood.

Experiments in rodents have identified some of the key inputs that control T and B cell homeostatic responses to lymphopenia via transfer of mature T or B cells into lymphopenic recipients (1–3). For mature T cells, homeostatic proliferation is regulated by IL-7 and IL-15 as well as by the interaction of the T cell receptor with self-MHC/peptide complexes and costimulatory signals (3–5). For mature B cells, the response to lymphopenia is independent of T cells, and it is inhibited by the presence of naive mature B cells as a function of B cell pool size (1, 6, 7). Likely regulators of the response to lymphopenia include tonic signaling through the B cell receptor (BCR) and the cytokine known as B cell activating factor (BAFF), with critical sources of BAFF identified among nonhematopoietic fibroblastic stromal cells (8, 9). BCR and BAFF-derived signals cooperate through shared intermediates, including Syk and members of the noncanonical NF-κB signaling pathway (10, 11). Consistent with the notion that the size of the peripheral B cell pool is not determined by BM production rates (7), BAFF has no impact on BM B cell progenitors but profound effects on transitional and mature B cell subsets in the periphery (12, 13). BAFF levels and availability inversely correlate with the degree of B cell lymphopenia, providing one mechanism through which available B cell space can be sensed by B cells (14). Thus, mature B cells respond to the degree of lymphopenia in their microenvironment, although the mechanisms and spatial regulation controlling this proportional response remain largely ill-defined.

Lymphopenia induces changes in the composition and differentiation state of naive mature B cells that are not only based on a shift in the balance between survival and proliferation. In mice, newly formed splenic transitional B cells differentiate into 2 major naive B cell subsets, follicular B (FoB) cells and marginal zone B (MZB) cells (15–17). Analogous populations have been identified in human lymphoid tissues, although with less granularity than in mice (18). In contrast to FoB cells, which represent the most abundant B cell subset in secondary lymphoid organs, MZB cells exhibit innate-like characteristics, unique positioning in the spleen close to incoming bloodborne antigens, and rapid responsiveness to stimuli leading to plasma cell differentiation, all consistent with their participation in a first line of defense. Importantly, in experimental conditions that abolished de novo B cell production, mature FoB cells became progressively depleted while numbers of MZB cells were maintained (19). Furthermore, elevated BAFF levels increased relative and absolute MZB cell numbers (20, 21). Thus, the current understanding favors the view that adaptive responses to B cell lymphopenia differentially affect naive B cell compartments and favor retention of innate-like B cells.

In steady-state conditions, development of MZB cells relies on Notch signaling mediated by the interaction of Notch2 receptors in B cells with Delta-like 1 (Dll1) Notch ligands in nonhematopoietic fibroblastic stromal cells (22–24). The effects of Notch signaling on MZB cells are exquisitely dosage-sensitive, as shown by haploinsufficiency of multiple critical genes in the pathway (22–26). Emerging evidence indicates that Notch signaling is not only required for the development of MZB cells, but that continuous Notch signals are essential for their maintenance and function (27, 28). Furthermore, induced expression of a gain-of-function Notch allele in FoB cells drove their differentiation to a MZB cell fate, showing that plastic changes in differentiation can be driven by Notch, at least under artificial conditions (29). Yet, it is unknown if Notch signals are engaged physiologically in response to lymphopenic environments, and if/how B cells integrate Notch with other signals in their homeostatic responses to B cell lymphopenia.

In this report, we explored the mechanisms coupling the differentiation and homeostatic proliferation of naive mature B cells in lymphopenic environments. Using an adoptive transfer model in which highly purified FoB cells were introduced into recipients with either normal lymphocyte numbers, selective loss of MZB cells, or profound lymphopenia, we report that FoB cells rapidly acquired multiple phenotypic, functional, and molecular characteristics of MZB cells in lymphopenic hosts. Lymphopenia-induced changes in differentiation preceded a burst of cell division, consistent with a process of B cell transdifferentiation taking place ahead of homeostatic proliferation. Mechanistically, Dll1/Notch2-mediated signals were essential for lymphopenia-induced B cell transdifferentiation, for their proliferation, and for their subsequent differentiation into antibody-producing plasma cells. Among potential cellular sources of Dll1, rare fibroblastic stromal cells lineage-traced with a *Ccl19-Cre* transgene were the dominant source of Notch ligands to support B cell transdifferentiation and proliferation. Thus, stromal Notch ligands emerge as a limiting resource that B cells compete for in lymphoid tissues. In turn, increased access to stromal Notch ligands in lymphopenic niches represents a critical spatial checkpoint to control the magnitude and functional consequences of B cell homeostatic responses to lymphopenia.

## Results

### Acquisition of a MZB cell–like phenotype precedes proliferation of FoB cells in lymphopenic hosts.

**Figure 1:**
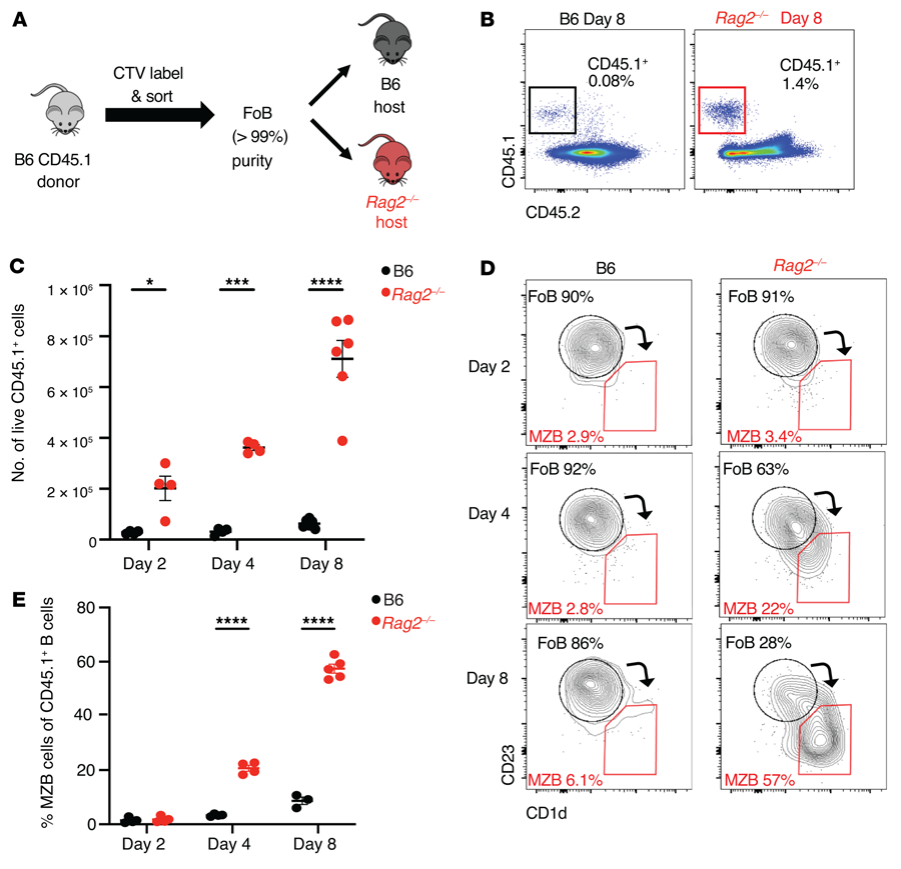
Follicular B cells adoptively transferred into lymphopenic hosts progressively acquire a marginal zone B cell phenotype. Highly purified congenically marked B6-CD45.1^+^ FoB cells were adoptively transferred (i.v.) into B6 or *Rag2^–/–^* hosts. Flow cytometry was performed on recipient spleen on days 2, 4, and 8 after transfer. (**A**) Experimental model. (**B**) Flow cytometric identification of donor-derived CD45.1^+^ cells recovered from B6 and *Rag2^–/–^* mice 8 days after transfer, gated on single live lymphocytes. (**C**) Absolute number of CD45.1^+^ donor-derived lymphocytes at days 2, 4, and 8 after transfer in B6 (black) or *Rag2^–/–^* (red) recipients. Data shown as mean ± SEM. (**D**) Analysis of adoptively transferred CD45.1^+^ FoB cells 2, 4, and 8 days after transfer by CD23 and CD1d expression in B6 (left) and *Rag2^–/–^* (right) recipients. Arrows depict shifts in cell surface phenotype. (**E**) Percentage of CD45.1^+^ cells that acquired a MZB cell phenotype by days 2, 4, and 8. Each data point represents an individual mouse. *P < 0.05, ***P < 0.001, and ****P < 0.0001, by 2-way ANOVA.

**Figure 2:**
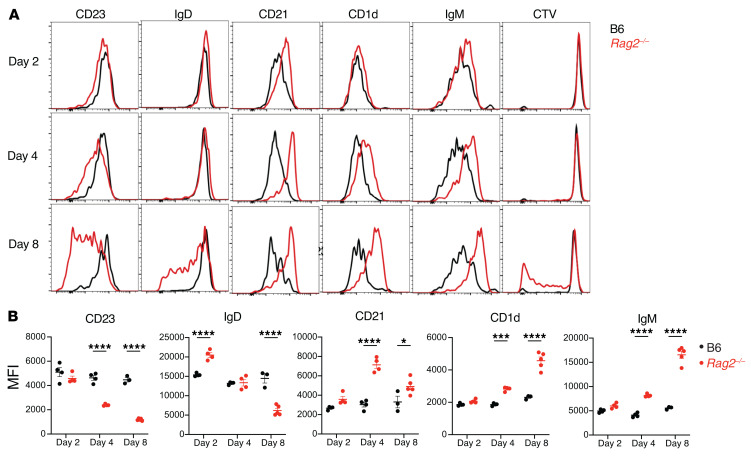
Follicular B cells transferred into lymphopenic hosts acquire a marginal zone B cell phenotype and then proliferate. Highly purified congenically marked B6-CD45.1^+^ FoB cells were labeled with CTV and adoptively transferred (i.v.) into B6 or *Rag2^–/–^* hosts. Flow cytometry was performed on recipient spleen on days 2, 4, and 8 after transfer. (**A**) Histograms depicting cell surface expression of markers and CTV dilution in CD45.1^+^ B cells recovered from B6 (black) and *Rag2^–/–^* (red) recipients. One representative sample of 3–4 mice is shown. (**B**) Quantification of the MFI for the indicated markers. Each data point represents an individual mouse. *P < 0.05, ***P < 0.001, and ****P < 0.0001, by 2-way ANOVA.

To evaluate how mature B cell populations respond to lymphopenia, we adoptively transferred FoB cells into *Rag2^–/–^* lymphopenic mice, which lack mature B and T cells (Figure 1A). Using B6-CD45.1 mice as donors, we isolated FoB cells to a high degree of purity and labeled them with CellTrace Violet (CTV) proliferation dye. We then transferred these cells into either B6-CD45.2 (B6) lymphoid-replete hosts or B6-*Rag2^–/–^* (*Rag2^–/–^*) lymphopenic recipients. We tracked donor-derived CD45.1^+^ cells at different time points after transfer in spleen and lymph nodes, characterized them using flow cytometry, and compared their phenotype with that of normal FoB and MZB cells (Figure 1, B–E; Figure 2; and Supplemental Figure 1, A–C; supplemental material available online with this article; https://doi.org/10.1172/JCI158885DS1).

At days 2, 4, and 8 after transfer, we recovered higher relative and absolute numbers of CD45.1^+^ donor-derived cells from the spleens of *Rag2^–/–^* as compared with B6 recipients (Figure 1, B and C). Increased B cell recovery was not associated with evidence of decreased apoptosis of the transferred cells in lymphopenic recipients, as annexin V staining was instead moderately increased among donor-derived B cells in *Rag2^–/–^* as compared with B6 hosts (Supplemental Figure 1D). These data suggested higher uptake, retention, and/or homeostatic expansion of FoB cells in the lymphopenic spleen environment.

Analysis of the spleens at day 2 after transfer showed that the majority of adoptively transferred cells retained the cell surface phenotype of FoB cells in both B6 and *Rag2^–/–^* hosts (Figure 1, D and E, and Figure 2), although some of the cells started to upregulate expression of surface IgM (sIgM) and CD21 in lymphopenic recipients (Figure 2). By day 4, donor-derived B cells had upregulated CD21, CD1d, and sIgM and downregulated CD23 expression in lymphopenic hosts, with approximately 20% now showing a CD1d^hi^CD23^lo^ phenotype reminiscent of MZB cells in *Rag2^–/–^* but not B6 recipients (Figure 1, D and E; Supplemental Figure 1, B and C; Figure 2). By day 8, the majority of donor cells transferred into *Rag2^–/–^* hosts but not B6 hosts acquired a MZB cell phenotype with reduced expression of CD23 and IgD and upregulated expression of CD21, CD1d, and sIgM at levels similar to wild-type MZB cells. Dilution of the CTV proliferation dye revealed a burst of proliferation between day 4 and day 8 in lymphopenic hosts, indicating that the early acquisition of MZB cell characteristics preceded proliferation (Figure 2 and Supplemental Figure 1, B and C). In lymph nodes, we recovered a higher fraction of donor-derived CD45.1^+^ cells in *Rag2^–/–^* as opposed to B6 recipients at day 8 after transfer, but B cells preserved their FoB phenotype both in B6 and *Rag2^–/–^* recipients (Supplemental Figure 1, E and F). These observations suggest that the splenic lymphopenic environment specifically supports transdifferentiation of FoB to MZB cells, followed by their proliferation.

### Transferred B cells cluster at the spleen marginal sinus in lymphopenic recipients.

**Figure 3:**
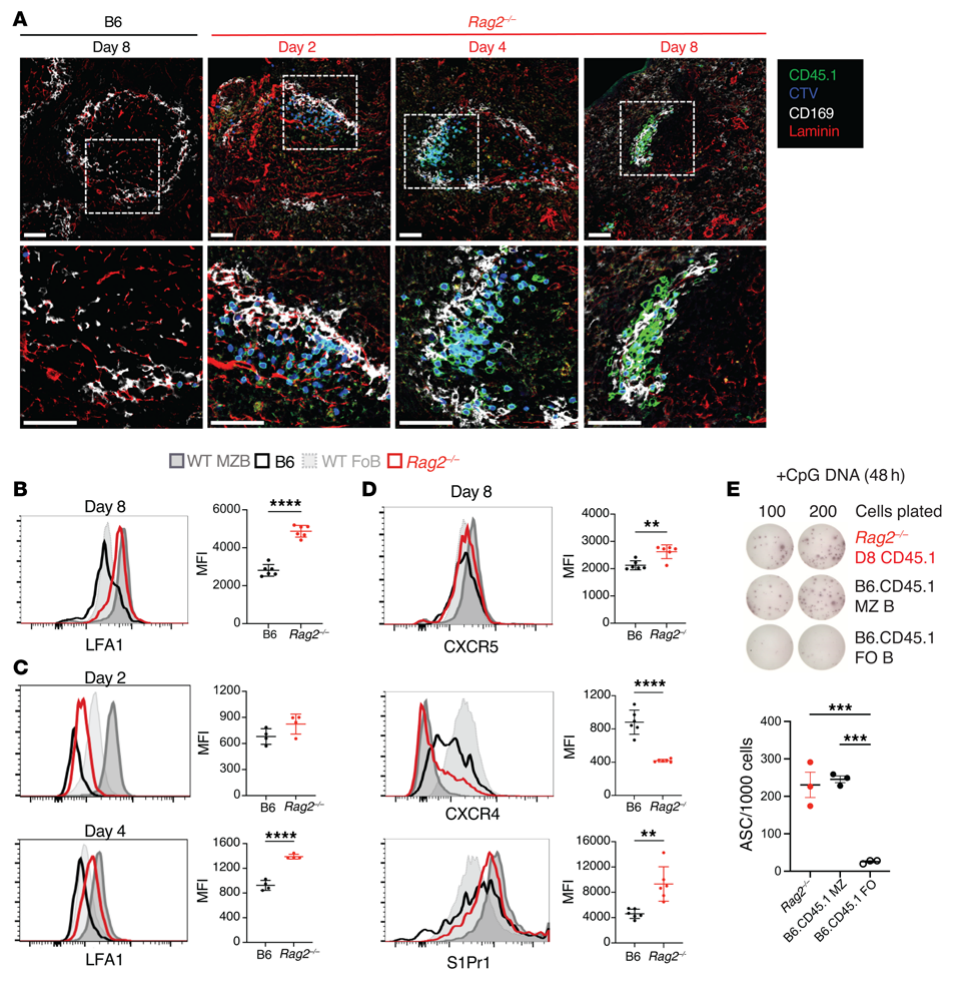
Follicular B cells transferred into lymphopenic hosts localize to marginal zone–like structures in splenic follicles and acquire integrin and chemoattractant receptor expression of marginal zone B cells. Congenically marked CD45.1^+^ FoB cells were labeled with CTV and adoptively transferred into B6 or *Rag2^–/–^* hosts. Flow cytometry and immunofluorescence microscopy were then performed on recipient spleens. (**A**) Immunofluorescence microscopy of spleen sections of B6 and *Rag2^–/–^* recipients at the indicated time points after adoptive transfer, with labeled CD45.1^+^ cells (green), CTV (blue), CD169 (white), and laminin (red). Proliferating cells that dilute CTV appear lighter in color. Dotted regions are magnified in the second row of images. One representative sample out of 4 is shown. Scale bars:50 μm. (**B**–**D**) Histograms depict the expression of LFA-1, S1Pr1, CXCR5, and CXCR4 among resting donor FoB or MZB cells from B6-CD45.1 donor mice (light/dark gray) or CD45.1^+^ B cells recovered from B6 (black) and *Rag2^–/–^* (red) recipients on day 8 after transfer (**B** and **D**) and on days 2 and 4 (**C**). Graphs quantify the MFI of indicated surface markers among CD45.1^+^ B cells recovered from B6 or *Rag2^–/–^* recipients. Data are shown as the mean ± SEM. **P < 0.01 and ****P < 0.0001, by 2-tailed Student’s *t* test. (**E**) Donor-derived CD45.1^+^CD19^+^ B cells recovered from *Rag2^–/–^* recipients at day 8 after transfer and freshly sorted B6-CD45.1 MZB and FoB cells were stimulated in culture with CpG DNA for 2 days. Antibody secretion from live cells was assessed by ELISpot. Representative wells are displayed and the number of antibody-secreting cells (ASCs) per 1000 live cells in culture is shown (individual data points indicate the mean ± SEM). ***P < 0.001, by 1-way ANOVA, Tukey’s post test. (**B**–**E**) Each data point represents an individual mouse.

One of the key characteristics of MZB cells is their location in the splenic marginal zone (MZ), a strategic positioning carefully regulated by integrins and chemoattractant receptors (30–33). To examine the distribution of adoptively transferred cells, we performed immunofluorescence imaging in spleen sections from recipient mice in the days following adoptive transfer (Figure 3A). Staining for CD169 identified metallophilic macrophages associated with the marginal sinus both in B6 and in *Rag2^–/–^* recipients. In B6 hosts, few CD45.1^+^ donor-derived cells were found scattered throughout the follicle, and most of them retained their CTV label, consistent with absence of proliferation. In contrast, *Rag2^–/–^* hosts displayed most CD45.1^+^ donor-derived B cells clustered around CD169^+^ macrophages in areas resembling an MZ, although the majority of transferred B cells were associated with the inner layer of the macrophage rim (facing white rather than red pulp). Most B cells remained CTV-positive at days 2 and 4 but lost their CTV label by day 8, consistent with a proliferation burst between day 4 and day 8. Thus, early B cell clustering around the marginal sinus of *Rag2^–/–^* recipients did not result from local proliferation.

We then analyzed surface expression of chemoattractant receptors and integrins in donor-derived B cells. At days 4 to 8 after transfer, B cells transferred into *Rag2^–/–^* but not into B6 hosts showed upregulated expression of LFA-1, a heterodimeric integrin essential for the retention of normal MZB cells and expressed at higher levels in MZB than FoB cells (Figure 3, B and C). Cell surface abundance of S1PR1 and CXCR5, which are critical for the positioning and shuttling of MZB cells between the MZ and the follicle (30, 32, 33), was also upregulated in B cells transferred to *Rag2^–/–^* recipients (Figure 3D). At the same time, B cells from *Rag2^–/–^* hosts downregulated surface CXCR4, a chemokine receptor whose expression is normally repressed in MZB cells compared with FoB cells. Next, we studied the ability of donor-derived B cells recovered from *Rag2^–/–^* recipients to rapidly produce antibodies when stimulated ex vivo with CpG only (a response mimicking T cell–independent differentiation and a key functional property of MZB but not FoB cells). Indeed, MZB cells are characterized by a preactivated state and by their capacity to produce plasma cells more rapidly than FoB cells, with reduced requirements for prior division and for exposure to cytokines (28, 34). In ELISpot assays, CpG stimulation of CD45.1^+^ B cells recovered at day 8 from *Rag2^–/–^* recipients induced a high frequency of antibody-secreting cells, similar to that observed with normal MZB cells and much higher than with FoB cells (Figure 3E). Altogether, transdifferentiated B cells in lymphopenic mice mimicked key positional and functional characteristics of normal MZB cells.

### Transdifferentiating B cells give rise to antibody-producing plasma cells in vivo.

**Figure 4:**
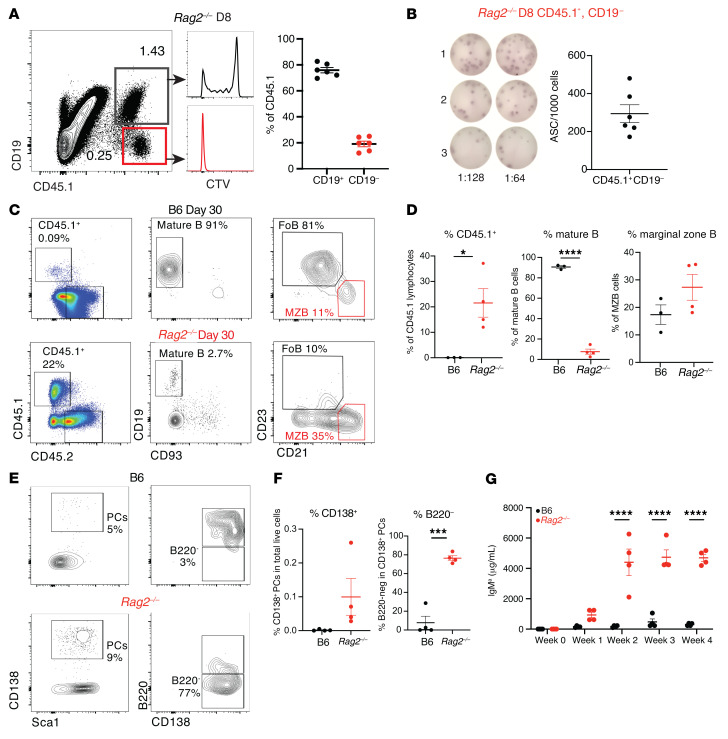
Transdifferentiated B cells give rise to functional plasma cells in lymphopenic mice. Congenically marked B6-C20-CD45.1^+^ FoB cells (Igh^a^) were adoptively transferred into B6 or *Rag2^–/–^* hosts. (**A**) CTV-labeled CD45.1^+^ donor-derived cells were assessed at day 8 after transfer for CD19 expression and cell division (CTV dilution). (**B**) CD45.1^+^CD19^–^ cells sorted from *Rag2^–/–^* recipient spleens at day 8 after transfer were plated in a serial dilution ELISpot assay. Numbers of CD45.1^+^ cells sorted by CD19 expression in each mouse are shown with representative ELISpot wells. Each data point represents an individual mouse. (**C**) Flow cytometry performed on recipient splenocytes 30 days after transfer. CD45.1^+^ mature FoB and MZB-like cell populations as detected in the spleen of B6 (top) and *Rag2^–/–^* (bottom) adoptive transfer recipients on day 30. One representative of 4 is shown. (**D**) Percentages of CD45.1^+^ lymphocytes, CD19^+^ B cells, and MZB cells, respectively. Data shown as mean ± SEM. (**E**) CD138^+^Sca-1^+^ plasma cells (PCs, left) and B220^+^CD138^+^ and B220^–^CD138^+^ PCs (right) detected in B6 and *Rag2^–/–^* recipient spleens at day 30 after transfer. (**F**) Percentages of CD138^+^ total PCs (left) and B220^–^ PCs (right). (**G**) Quantification of donor-derived serum IgM^a^ concentration in B6 (black) and *Rag2^–/–^* (red) recipients at the indicated time points after transfer. (**D**, **F**, and **G**) *P < 0.05, ***P < 0.001, and ****P < 0.0001, by 2-way ANOVA. Each data point represents an individual mouse.

To test whether newly formed MZ-like B cells could rapidly differentiate into antibody-secreting cells in vivo, we first assessed the emergence and function of donor-derived cells downregulating CD19 expression after FoB cell transfer to *Rag2^–/–^* recipients. At day 8 after transfer, approximately 20% of donor-derived cells had lost CD19 expression and had fully diluted the CTV label, indicating an extensive proliferation history (Figure 4A). We sort-purified these CD19^–^ cells and cultured them directly in ELISpot plates without any ex vivo stimulation. Interestingly, approximately 30% of these cells were functional antibody-secreting cells, suggesting that donor-derived MZ-like B cells could further differentiate into antibody-secreting plasma cells in lymphopenic environments (Figure 4B).

We then investigated cellular features of plasma cell differentiation among donor-derived cells at day 30 after transfer. We recovered very few CD45.1^+^ donor cells from the spleens of lymphoid-replete B6 hosts, most of which exhibited high CD19 expression. Although approximately 10% had differentiated into MZB cells, the majority preserved a FoB cell phenotype (Figure 4, C–F). In contrast, we recovered a higher percentage of CD45.1^+^ donor-derived cells in the spleens of *Rag2^–/–^* hosts at day 30, most of which had lost CD19 expression. Among the remaining CD19^+^ cells, the majority retained a MZB cell phenotype. Many donor-derived CD138^+^ plasma cells arose in lymphopenic but not B6 hosts, most of them with a B220^lo^ long-lived plasma cell phenotype (Figure 4, E and F). To assess whether donor-derived B cells gave rise to in vivo antibody production in *Rag2^–/–^* recipients, we used donor mice expressing the Igh^a^ allotype to allow tracking of serum antibodies produced by donor-derived cells. We purified FoB cells from C57BL/6-C20-CD45.1 donor mice expressing Igh^a^ and adoptively transferred them into *Rag2^–/–^* or B6 hosts expressing Igh^b^. We quantified serum IgM^a^ antibodies at different time points after transfer as a reflection of donor-derived antibody production (Figure 4G). We detected increased levels of donor-derived IgM^a^ antibodies in *Rag2^–/–^* hosts by day 7 with a persistent peak starting at day 14 after transfer, whereas very low levels of IgM^a^ were detected in B6 hosts. Therefore, transdifferentiated B cells exposed to a lymphopenic environment were endowed with a plasma cell differentiation potential that could contribute to long-lasting in vivo antibody production.

### FoB cells acquire a full MZB cell transcriptional program in lymphopenic recipients and rapidly upregulate a broad Notch signature.

**Figure 5:**
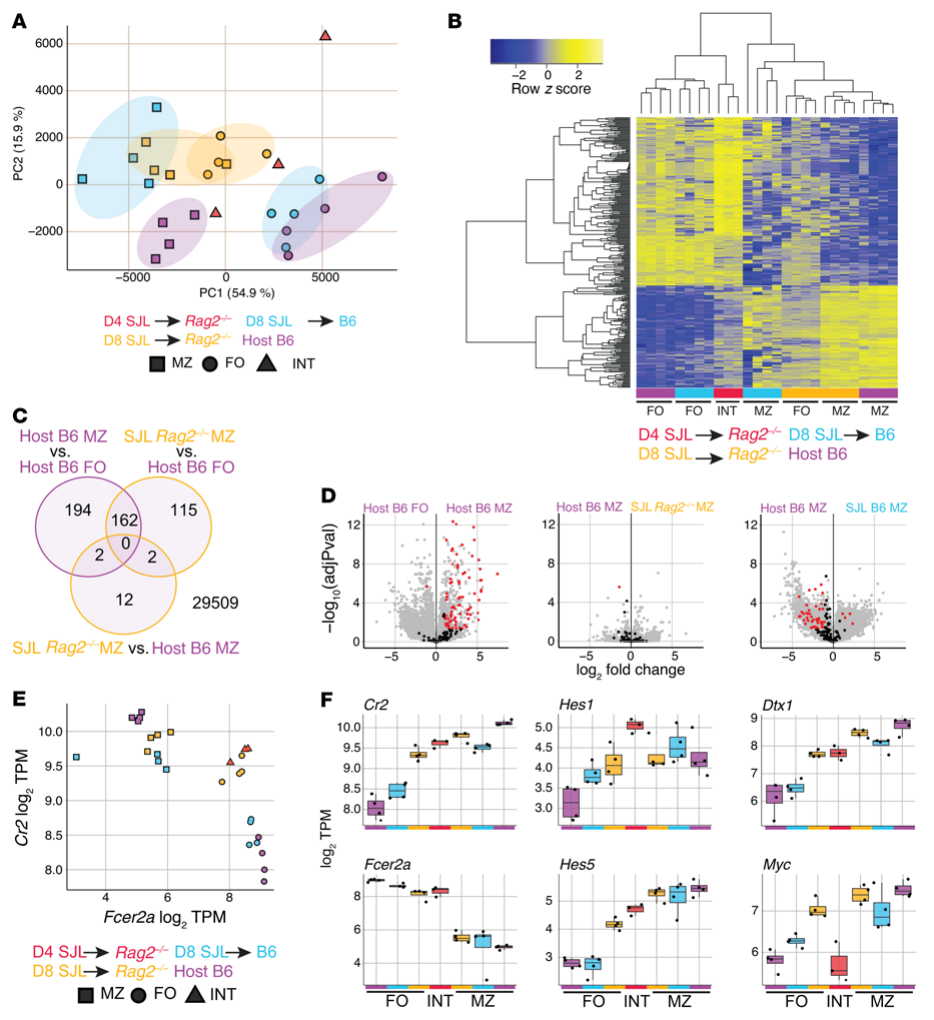
Follicular B cells adoptively transferred into a lymphopenic environment attain a full marginal zone B cell transcriptome and upregulate a broad Notch signature. Congenically marked B6-CD45.1 FoB cells were labeled with CTV and i.v. transferred to either B6 or *Rag2^–/–^* hosts. RNA-Seq was then performed on B cells twice-sorted from animals euthanized after 4 and 8 days, sorting first on CD45.1/2 and second on follicular or marginal zone B cell gates (d8) or intermediate gates (d4) (see Supplemental Figure 2). (**A**) Principal component analysis of all samples with colors indicating transfer groups and times and shapes indicating cell-type sort gates. (**B**) Expression of a MZB/FoB signature — defined as genes differentially expressed between host B6 MZB and FoB cells (adjusted *P* value < 0.01, log_2_ fold change > 2) — is shown for all samples as the *z* score across each row. Samples in columns are hierarchically clustered by Spearman’s correlation. (**C**) Venn diagrams displaying differential gene testing results of the indicated comparisons. Shown are the number of genes differential (adjusted *P* value < 0.01, log_2_ fold change > 2) without respect to direction. (**D**) Volcano plots indicating the magnitude and significance of gene expression changes between indicated groups for all (gray), and highlighted (black/red) empirically defined MZB cell Notch2-regulated genes (genes significantly downregulated in MZB cells after 24 hours of anti-N2 antibody blockade) (28). Red indicates significance (adjusted *P* < 0.05; log_2_ fold change > 1). (**B**–**D**) Differential expression was calculated by empirical Bayes method with Benjamini-Hochberg correction with indicated cutoffs. (**E** and **F**) Log_2_ transcripts per million (TPM) are shown for indicated genes as 2-gene correlation (**E**) and selected individual genes (**F**). Adoptive transfer groups are shown by color, and sort gate is indicated by shape.

To evaluate the MZB cell differentiation state of adoptively transferred B cells beyond their surface phenotype, we next examined the transcriptome of these cells. We performed RNA-Seq on twice-sorted donor-derived B cells 4 and 8 days after transfer into *Rag2^–/–^* and B6 recipients, as well as from host-derived B cells from B6 animals (as representative normal B cell subsets). At day 4 after transfer, we purified donor-derived B cells from a population with intermediate phenotypic characteristics. At day 8, FoB-like and MZB-like cells were sorted separately (Supplemental Figure 2A). Principal component analysis clustered each sample type and broadly separated FoB and MZB cell groups across PC1 (59% of variation), and day 4 intermediate population cells fell between the 2 groups (Figure 5A). Interestingly, donor-derived cells sorted with FoB cell gates from *Rag2^–/–^* recipients at day 8 clustered closer to normal MZB cells than to normal FoB cell populations, suggesting induction of a MZB-like transcriptional state even with residual FoB phenotypic features.

We then prepared a MZB/FoB cell signature defined by genes differentially expressed in B6 MZB versus FoB cells and hierarchically clustered all samples based on this signature (Figure 5B). Both donor-derived day 4 intermediate and day 8 donor-derived FoB-like cells purified from *Rag2^–/–^* recipients showed intermediate expression across the MZB/FoB signature, consistent with partial transdifferentiation at this stage. At day 8, donor-derived cells sorted as MZB cells in *Rag2^–/–^* recipients clustered tightly with B6 MZB cells with very few differentially expressed genes, indicating that lymphopenia-induced transdifferentiation fully reprogrammed FoB cells to a MZB cell state (Figure 5, B and C). Interestingly, donor-derived MZB cells in B6 recipients had more differentially expressed genes compared with both B6 MZB cells and donor-derived MZB cells in *Rag2^–/–^* recipients, suggesting that lymphopenia enhanced the acquisition of the MZB cell transcriptional program in B cells (Figure 5B and Supplemental Figure 2B).

We then investigated whether donor-derived B cells displayed evidence of Notch signaling, as well as the kinetics and extent of Notch-regulated gene expression in the presence or absence of lymphopenia (Figure 5, D and E, and Supplemental Figure 2C). Normal MZB cells depend on Notch2-mediated signals for their development as well as for their maintenance and function (22, 27, 28). We used a gene signature that we previously identified as downregulated in steady-state MZB cells after 24 hours of in vivo antibody-mediated Notch2 inhibition, in comparison with that of B6 FoB and MZB cells (28). Donor-derived MZB cells sorted from *Rag2^–/–^* recipients displayed upregulation of Notch-regulated gene expression, and the degree of upregulation was even larger than observed in normal B6 MZB cells as compared with FoB cells (suggesting the occurrence of intense Notch signals in B cells exposed to lymphopenia). In contrast, donor-derived MZB cells in B6 recipients showed incompletely upregulated expression of these genes, consistent with more efficient induction of Notch signaling in lymphopenia (Figure 5D and Supplemental Figure 2C). Donor FoB-like cells recovered from *Rag2^–/–^* recipients also had significant upregulation of Notch-regulated gene expression as compared with normal B6 FoB cells, suggesting that FoB cells were rapidly subjected to high Notch signaling intensity when transferred to lymphopenic hosts, even before differentiating into phenotypically defined MZB cells (Supplemental Figure 2C).

Transcript abundance for *Cr2* (encoding CD21, a Notch target gene) and *Fcer2a* (encoding CD23) paralleled CD21 and CD23 protein expression as used for purification, with the exception of day 4 intermediate cells and day 8 FoB-like cells from *Rag2^–/–^* recipients that had intermediate levels of these transcripts (Figure 5E). All donor-derived B cell subsets recovered from *Rag2^–/–^* as compared with B6 recipients (including FoB-like cells) showed increased expression of the Notch target genes *Cr2*, *Dtx1*, *Hes1*, and *Hes5*, similar to host MZB cells, consistent with the early induction of a Notch signature upon exposure of FoB cells to lymphopenic niches. Interestingly, *Myc* expression was not increased in day 4 sorted intermediate cells from *Rag2^–/–^* animals, despite *Myc* being a conserved Notch target in normal and malignant B cells (Figure 5F and refs. 28, 35). Thus, *Myc* induction was delayed in B cells compared with other Notch target genes, coinciding with an equally delayed onset of proliferation. Together, these data indicate that FoB cells transferred into lymphopenic mice efficiently interacted with Notch ligands, adopting a MZB cell phenotype and a full MZB cell transcriptomic state. This phenomenon was magnified and accelerated in lymphopenic as compared with lymphoid-replete mice, suggesting increased access to Notch ligands during the B cell response to lymphopenia.

### Dll1-Notch2 interactions are essential to support B cell transdifferentiation and proliferation in lymphopenic mice.

**Figure 6:**
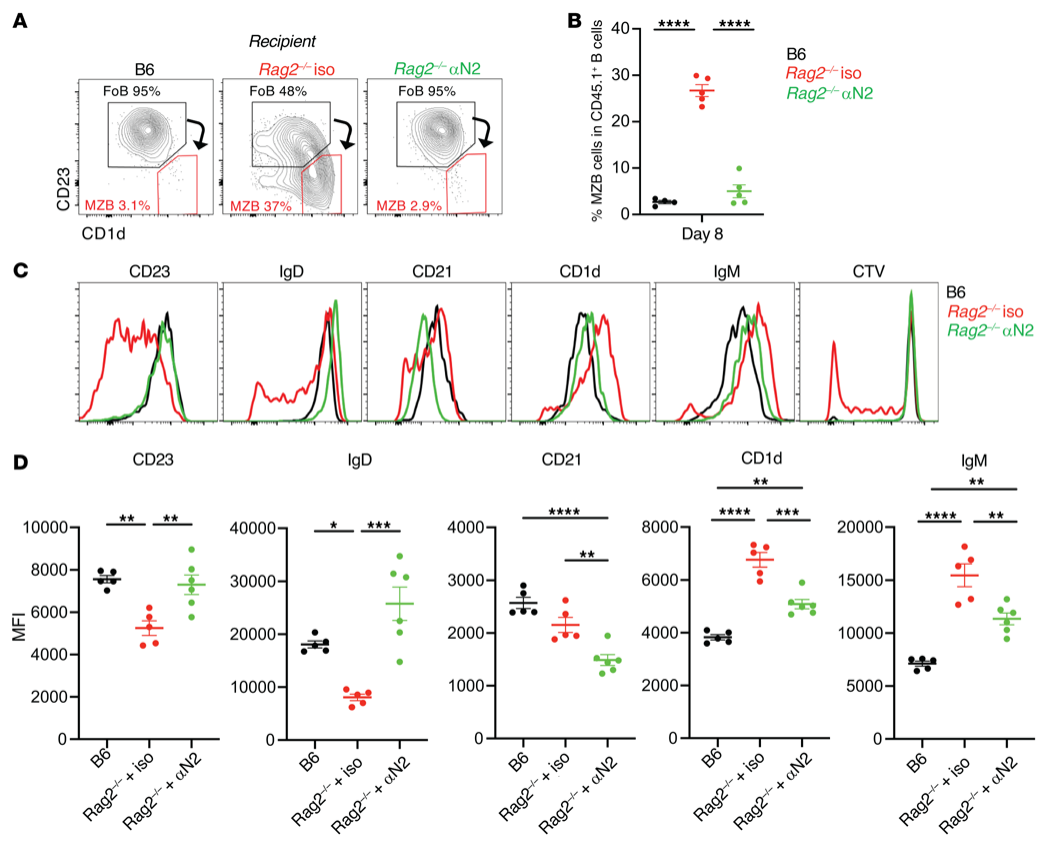
Notch2 signals are required for the transdifferentiation of follicular B cells into marginal zone–like B cells in lymphopenic recipients. Congenically marked CD45.1^+^ FoB cells were labeled with CTV and adoptively transferred into B6 or *Rag2^–/–^* hosts treated with isotype control or anti-Notch2 antibodies on days 0, 3, and 6 after transfer. (**A**) Representative flow cytometry plots depicting live CD45.1^+^CD19^+^CD93^–^ donor-derived mature B cells recovered at day 8 from B6 or indicated *Rag2^–/–^* recipients. (**B**) Percentage of CD45.1^+^ MZB cells at day 8 in each recipient group. Data shown as mean ± SEM. (**C**) Histograms depicting cell surface expression of markers and CTV dilution among donor-derived CD45.1^+^ B cells in B6 (black), *Rag2^–/–^* recipients treated with isotype control antibodies (Iso, red), or *Rag2^–/–^* recipients treated with anti-Notch2 antibodies (green). Results are representative of 2 independent experiments. (**D**) Quantification of the MFI for the indicated markers. (**B** and **D**) *P < 0.05, ***P < 0.001, and ****P < 0.0001, by 1-way ANOVA. Each data point represents an individual mouse.

We next assessed functionally whether FoB transdifferentiation into MZ-like B cells and their subsequent proliferation in lymphopenic environments required Notch signaling. We transferred FoB cells into B6 or *Rag2^–/–^* recipients treated either with an isotype control or an anti-Notch2 antibody, which efficiently blocks ligand-induced cleavage and activation of Notch2 receptors in vivo (28, 36, 37). In *Rag2^–/–^* hosts that received anti–Notch2 as compared with control antibodies, phenotypic changes associated with transdifferentiation of FoB cells into MZ-like B cells were completely abrogated (Figure 6, A–D). Anti-Notch2 antibodies blocked upregulated expression of CD21 encoded by the *Cr2* gene, a conserved direct transcriptional target of Notch signaling (35, 38, 39). Notch2 inhibition also blocked CD1d upregulation, CD23 and sIgD downregulation, and subsequent homeostatic proliferation. Indeed, transferred FoB cells preserved their original phenotype just as in lymphoid-replete B6 recipients. Thus, both B cell transdifferentiation and homeostatic proliferation in lymphopenic spleens required Notch2 signaling.

**Figure 7:**
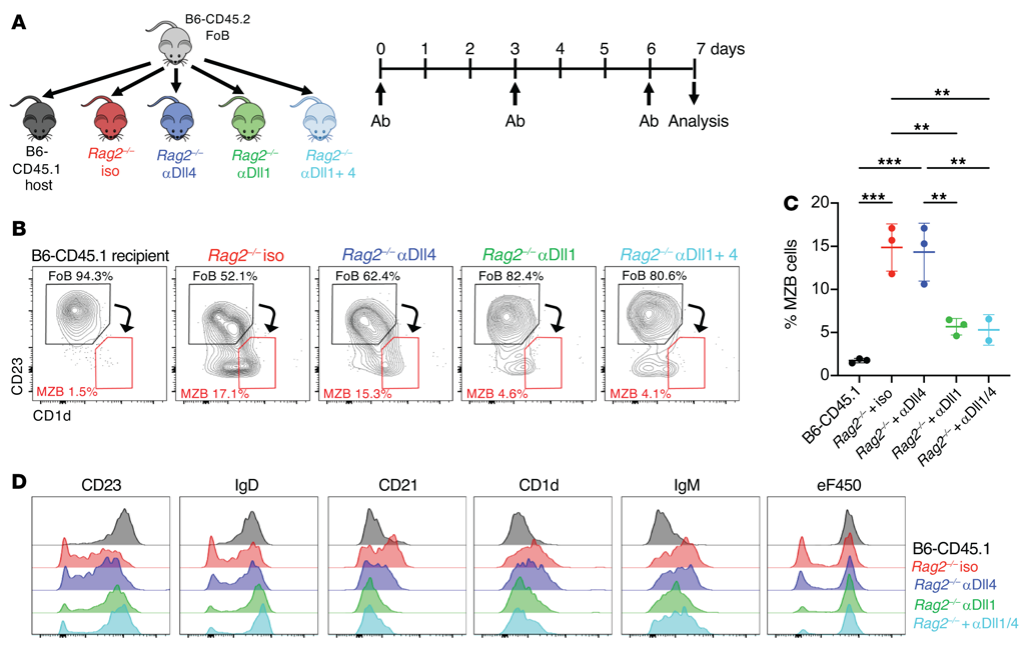
B cell homeostatic responses to lymphopenia depends on Delta-like 1 Notch ligands. Congenically marked CD45.2^+^ FoB cells were labeled with eF450 and adoptively transferred into B6-CD45.1 or *Rag2^–/–^* recipients treated with isotype control or anti-Dll1, anti-Dll4, or both antibodies at days 0, 3, and 6 after transfer. Flow cytometric analysis of recipient spleens was performed at day 8 after transfer. (**A**) Experimental model. (**B**) Representative flow cytometry plots gated on CD45.2^+^CD19^+^CD93^–^ live donor-derived B cells recovered at day 8 from B6-CD45.1 mice or CD19^+^CD93^–^ B cells from indicated *Rag2^–/–^* recipient groups. (**C**) Percentage of donor-derived marginal zone–like B cells at day 8 in each recipient group. Each data point represents an individual mouse. Data shown as mean ± SEM. (**D**) Histograms depicting cell surface expression of the indicated markers and eF450 dilution among donor-derived B cells from indicated B6-CD45.1 or *Rag2^–/–^* recipients. **P < 0.01 and ****P < 0.0001, by 1-way ANOVA.

Next, we evaluated which Notch ligands drive Notch2-mediated signaling in B cells exposed to lymphopenia. Normal MZB cells rely on the interaction of Notch2 receptors with Dll1 Notch ligands, although Dll4 Notch ligands are also expressed in the spleen and could become engaged in the setting of lymphopenia (24, 40). To address this question, we transferred FoB cells into *Rag2^–/–^* recipients treated with anti-Dll1 and/or anti-Dll4 antibodies, which specifically inhibit Dll1 or Dll4 in vivo (Figure 7A and refs. 37, 41). Anti-Dll1 antibodies profoundly inhibited all phenotypic changes and subsequent proliferation induced by lymphopenia in FoB cells. In contrast, Dll4 blockade alone had no impact on transdifferentiation and proliferation of FoB cells in *Rag2^–/–^* recipients and did not augment the effects of anti-Dll1 antibodies (Figure 7, B–D). Thus, Dll1-Notch2 interactions were the major drivers of the Notch-mediated B cell response to lymphopenia.

### Dll1-expressing Ccl19-Cre^+^ stromal cells drive the B cell response to lymphopenia.

**Figure 8:**
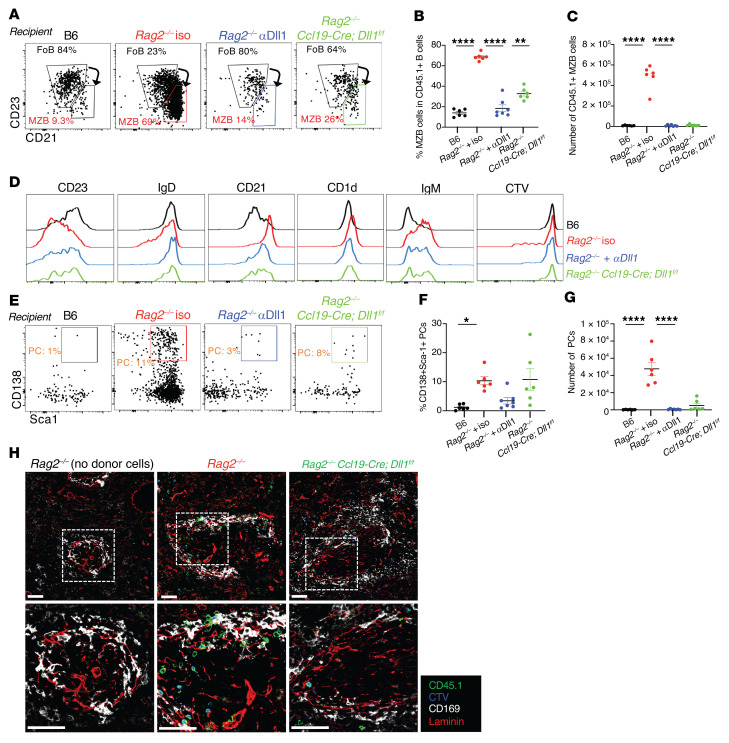
Stroma-derived Dll1 Notch ligands drive B cell transdifferentiation and plasma cell differentiation in lymphopenic recipients. B6 versus *Rag2^–/–^* versus *Rag2^–/–^* crossed to *Ccl19-Cre Dll1^fl/fl^* hosts were treated with isotype control or anti-Dll1 antibodies at days 0, 3, and 6 after transfer of purified B6-CD45.1 FoB cells. Flow cytometry and immunofluorescence imaging were performed on recipient splenocytes at day 8 after transfer. (**A**) Representative flow cytometry plots depicting live CD45.1^+^CD19^+^CD93^–^ donor-derived mature B cells recovered at day 8 from B6 or indicated *Rag2^–/–^* recipients. (**B**) Percentage and (**C**) absolute numbers of CD45.1^+^ MZB cells at day 8 in each recipient group. (**D**) Histograms depicting cell surface expression of markers and CTV dilution on donor derived CD45.1^+^ B cells in B6 (black), *Rag2^–/–^* (red), or indicated *Rag2^–/–^* recipients. (**E**) Representative flow cytometry plots showing live donor-derived CD138^+^ plasma cells at day 8 in B6 or indicated *Rag2^–/–^* recipients. (**F**) Percentage and (**G**) absolute numbers of donor-derived plasma cells at day 8 in each recipient group. (**H**) Immunofluorescence microscopy of spleen sections of B6, *Rag2^–/–^*, and *Rag2^–/–^* *Ccl19-Cre Dll1^fl/fl^* recipients at day 8 after adoptive transfer, with labeled CD45.1^+^ cells (green), CTV (blue), CD169 (white), and laminin (red). Dotted regions are magnified in the second row of images. Scale bars: 50 μm. Data are from 2 independent experiments. (**B**, **F**, and **G**) *P < 0.05, **P < 0.01, and ****P < 0.0001, by 1-way ANOVA. Each data point represents an individual mouse.

Past work in steady-state conditions identified a rare subset of nonhematopoietic fibroblastic stromal cells lineage-traced with a *Ccl19-Cre* transgene as the critical source of Dll1 for MZB cell homeostasis (24). Thus, we wondered to what extent these cells function as an immunological niche whose accessibility controls the B cell response to lymphopenia. To test this hypothesis, we crossed *Ccl19-Cre Dll1^fl/fl^* mice to a *Rag2^–/–^* background and adoptively transferred purified FoB cells into their progeny. Acquisition of a CD1d^hi^CD21^hi^CD23^lo^ MZB-like phenotype was profoundly inhibited in *Ccl19-Cre Dll1^fl/fl^ Rag2^–/–^* recipients as compared with *Rag2^–/–^* littermate recipients at day 8 after transfer (Figure 8, A–D). Ccl19-Cre–mediated *Dll1* inactivation blocked B cell transdifferentiation to a similar extent as systemic anti-Dll1 antibodies. We also analyzed the differentiation of transferred CD45.1^+^ B cells into plasma cells and found donor-derived plasma cells in the spleen of *Rag2^–/–^* hosts but not in *Rag2^–/–^* hosts treated with anti-Dll1 antibodies or upon *Dll1* inactivation in Ccl19-Cre^+^ stromal cells (Figure 8, E–G). Furthermore, donor-derived B cells lost their tight association with the marginal sinus in *Ccl19-Cre Dll1^fl/fl^ Rag2^–/–^* as compared with *Rag2^–/–^* hosts (Figure 8H). Thus, transdifferentiation of FoB cells into MZ-like B cells and subsequent plasma cell differentiation in lymphopenic environments required Dll1/Notch2 signals provided predominantly by a *Ccl19-Cre^+^* stromal cell niche.

To further evaluate the spatial requirements driving the response of B cells to stromal Notch ligands in the lymphopenic spleen, we tested whether a selective lack of B cells in the MZ was sufficient to support the transdifferentiation of adoptively transferred FoB cells into MZ-like B cells. To this end, we used *Mb1-Cre Notch2^fl/fl^* recipients that selectively lack MZB cells (due to the role of Notch2 in MZB cell homeostasis) but have a preserved FoB cell compartment. We compared the fate of FoB cells transferred into these mice with B6 or *Rag2^–/–^* recipients (Supplemental Figure 3, A and B). Interestingly, FoB cells underwent MZB cell transdifferentiation only in lymphopenic *Rag2^–/–^* hosts and not in B6 or in *Mb1-Cre Notch2^fl/fl^* recipients (Supplemental Figure 3C). These data indicate that access to a B cell–poor MZ compartment is not sufficient to support FoB-to-MZB cell transdifferentiation. Instead, we speculate that access to stromal Notch ligands in the FoB cell compartment is essential for the B cell response to lymphopenia and the subsequent acquisition of MZ-like B cell characteristics.

## Discussion

Our findings uncovered a central role for the interaction of mature B cells with an immunological stromal niche expressing Notch ligands during the adaptive response to B cell lymphopenia. Beyond an impact on homeostatic proliferation, Notch ligand/receptor interactions also controlled a full conversion of the B cell transcriptome to a distinct differentiation state, promoting the accumulation of innate-like B cells and their subsequent differentiation into functional plasma cells. The identification of membrane-bound Notch ligands in this niche represents a strictly spatially defined resource that controls the magnitude of the homeostatic response to lymphopenia. In turn, access to stromal Notch ligands emerges as a central mechanism through which the depth of lymphopenia can be sensed by B cells.

Notch ligands are membrane-bound proteins that signal through direct contact with receptors in adjacent cells. During B cell responses to lymphopenia, the dominant cellular source of Notch ligands was restricted to a specialized subset of fibroblastic stromal cells lineage-traced with a *Ccl19-Cre* transgene and previously reported to have immune-interacting functions in secondary lymphoid organs. Our findings identified a scenario in which naive mature B cells can probe the degree of lymphopenia in their environment as a function of their access to a limiting pool of Notch ligands in a noncirculating cell type, thereby defining an immunological niche similar in regulation and function to a stem cell niche. We propose that access to stromal Notch ligands in the FoB compartment is essential for the B cell response to lymphopenia because selective B cell depletion from the MZ was insufficient to support B cell transdifferentiation. These findings are consistent with the detection of Dll1 Notch ligands expressed by *Ccl19-Cre^+^* stromal cells within the FoB cell zone (24) and with past observations that MZB cells constantly shuttle between FoB and MZB cell compartments in the spleen (30).

Past work identified BAFF as a molecule that could partially explain the proportional response of B cells to lymphopenia, as BAFF levels and availability rise with low B cell numbers (14). Moreover, ablation of *Ccl19-Cre^+^* fibroblastic stromal cells revealed them to be the dominant source of BAFF in vivo (8). However, key effects of BAFF on transitional B cells and naive mature B cells can be achieved with systemic administration (21). Moreover, BAFF’s activity is profoundly impaired by mutation of its furin cleavage site, a proteolytic site necessary for the release of soluble BAFF (42). Thus, BAFF secretion may generate a local gradient, but it lacks the characteristics of a true anatomically restricted niche-associated factor. In contrast, Notch ligands emerge as the first membrane-bound molecule identified to provide spatial regulation during the B cell response to lymphopenia. These features may be particularly important to coordinate B cell pool size and spatial positioning. It remains to be explored how Notch cooperates with other signaling pathways involved in the B cell response to lymphopenia, including BAFF and BCR signaling. For example, beyond regulation through direct availability of Notch ligands in lymphopenic environments, it is possible that BAFF and/or BCR signaling increase Notch receptor expression or overall B cell sensitivity to Notch signaling.

Previous work identified plasticity in the FoB cell state (29, 43). Thus, the most abundant subset of naive B cells in secondary lymphoid organs is not locked in a terminal state of B cell differentiation ahead of antigen encounter. Gain-of-function studies using constitutively active Notch alleles revealed the potential of Notch signaling to drive the acquisition of a MZB cell fate when induced artificially in FoB cells (29). Our findings indicate that Notch2-driven B cell transdifferentiation can happen physiologically as a result of Notch ligand/receptor interactions during lymphopenia, and that these interactions represent a critical quantitative input through which mature B cells sense available B cell space. Lymphopenia rapidly exposes FoB cells to high levels of Notch signaling, which induces broad transcriptional, phenotypic, and functional changes consistent with the acquisition of a MZB cell fate. Because these effects were observed even before significant B cell proliferation was apparent, they are consistent with a process of transdifferentiation. Disruption of Jagged1/2-Notch2 interactions in mature lung epithelial cells was previously reported to induce transdifferentiation of secretory cells into ciliated cells in the complete absence of cell division, a departure from traditional models of Notch signaling controlling cell fate decisions in dividing multipotent progenitors (44). We speculate that a similar process is at play in mature B cells as a function of the intensity of Notch signals delivered by their environment. However, unlike the aforementioned transdifferentiation of lung epithelial cells, FoB-to-MZB cell transdifferentiation eventually becomes coupled to homeostatic proliferation of the transdifferentiating B cell pool.

Our transcriptional analysis indicates that phenotypic changes induced by lymphopenia in mature B cells can be mapped back to the conversion of their transcriptome to that of an alternative cell state, and not just to the differential expression of individual cell surface markers. Lymphopenia triggered a rapid and potent induction of a cohort of Notch-regulated genes in B cells. However, induction of *Myc* transcription represents an interesting exception, as it was delayed by several days after induction of most other canonical Notch target genes in B cells transferred to a lymphopenic environment. This was surprising given that *Myc* was identified as an evolutionarily conserved transcriptional Notch target in mouse MZB cells at steady state and in human Notch-driven mature B cell lymphomas (28, 35). Instead, the initial induction of *Myc* transcription may require additional inputs that cooperate with Notch signaling, including the expression of critical transcription factors or other regulators that control accessibility to and activity of the Notch-regulated enhancer in naive mature B cells. Functionally, we speculate that delayed *Myc* induction may represent a fail-safe mechanism so that mature B cells only commit to a burst of proliferation after prolonged exposure to a durable state of lymphopenia. In addition, this sequential response may be designed to prioritize early plasma cell differentiation and antibody production ahead of homeostatic expansion, thus providing rapid effector functions in reaction to an incoming threat.

Beyond the important function of stimulating B cell proliferation, the Notch-regulated B cell response to lymphopenia had a major functional impact on the B cell differentiation state, including subsequent spontaneous differentiation of the cells into antibody-producing plasma cells. The nature and specificity of the antibodies produced in this context remain to be determined. We speculate that this pattern of regulation was selected based on an evolutionary advantage to predominantly conserve innate-like B cells with rapid differentiation potential in situations of B cell scarcity (19). Conversely, the impact of Notch signaling could be significant in lymphopenic disease states, especially when increased selection of autoreactive B cells and production of pathogenic antibodies are important. For example, many autoimmune disorders are characterized by chronic lymphopenia as well as by the administration of lymphopenia-inducing treatments that could enhance B cell exposure to BAFF and to stromal Notch ligands. Similarly, these factors have been reported to affect pathogenic B cells in chronic graft-versus-host disease, a severe complication of allogeneic bone marrow transplantation in which B cell autoreactivity and antibody production play a significant pathogenic role (45, 46).

Altogether, emerging data highlight evolutionarily conserved functions of Notch in the B cell lineage. Indirect evidence for rapid replenishment of the MZB cell pool by mature B cells was first reported in lymphodepleted rats in classical experiments from MacLennan and colleagues (2). Our genetic and functional experiments in mice now situate Notch signaling at the core of this regulated adaptive response to lymphopenia. In humans, *NOTCH1* and *NOTCH2* function as recurrently mutated oncogenes in B cell lymphoproliferative disorders, including chronic lymphocytic leukemia and MZ lymphoma (35, 47–50). In this context, gain-of-function *NOTCH1/2* mutations nearly always retain ligand-dependence for Notch activation by increasing the half-life of cleaved intracellular Notch without affecting Notch receptor activation. Notch activation in malignant B cells can also be observed within but not outside lymphoid tissues in the absence of Notch pathway mutations, suggesting a role for microenvironmental sources of Notch ligands (47, 51, 52). Both in mouse B cells and in human B cell lymphomas, Notch controls a transcriptional module, including dozens of genes, which has been conserved during evolution (28, 35). We speculate that these functions of Notch signaling in the B cell lineage have been retained because of their fundamental importance in the interaction of B cells with their niche in secondary lymphoid organs and hijacked during transformation.

## Methods

### Mice.

C57BL/6 *Rag2^–/–^* (CD45.2, Ighb) mice were bred in-house. C57BL/6-CD45.2 (B6) (57BL/6J: strain 000664) and C57BL/6-CD45.1 (B6-CD45.1) (B6.SJL-Ptprca Pepcb/BoyJ: strain 002014) mice were purchased from The Jackson Laboratory. C57BL/6-C20 (Igh^a^) mice were originally obtained from Gayle Bosma (Institute for Cancer Research, Philadelphia, PA) and crossed to the B6-CD45.1 background (53). *Mb1-Cre* and *Notch2^fl/fl^* mice were described previously and intercrossed to inactivate *Notch2* in the B cell lineage, leading to the loss of MZB cells (54, 55). The *Ccl19-Cre* BAC transgene (driving Cre expression in Ccl19^+^ lymphoid tissue fibroblastic stromal cells) as well as the conditional floxed *Dll1* allele were described previously and were crossed to the *Rag2^–/–^* background (23, 40, 56).

### Antibodies, flow cytometry, and cell sorting.

Single-cell suspensions from the spleen or peripheral lymph nodes (cervical, brachial, axial, inguinal) were prepared by physical disruption through 40 μm cell strainers. Red cell lysis was performed using ACK lysis buffer (Quality Biological). Except when indicated, the following anti-mouse antibodies used for cell staining were obtained from BioLegend: anti-CD45.1 (clone A20), CD45.2 (clone 104), B220 (clone RA3-6B2), CD19 (clone 1D3), CD21 (clone 7E9), CD23 (clone B3B4), CD93 (clone AA4.1; eBioscience), CD138 (clone 281-2), CD1d (clone 1B1), sIgD (clone 11-26c.2a), IgM (clone RMM-1), Sca-1 (clone D7), LFA-1 (clone H155-78), S1PR1 (clone 713412; R&D Systems), CXCR5 (clone L138D7), and CXCR4 (clone L276F12). Dump gating for plasma cell staining was performed using antibodies for CD3 (clone 17A2), Ter-119 (clone TER119), and F4/80 (clone BM8). For S1PR1, staining was performed as previously reported (57). Briefly, charcoal-stripped FBS (Thermo Fisher Scientific) was used at 2% in DPBS with 1 mM EDTA. Cells were stained with rat anti-S1PR1 (clone 713412), followed by donkey anti-rat IgG-biotin F(ab’)2 (Jackson ImmunoResearch) and streptavidin-PE (BioLegend). Nonviable cells were excluded with Zombie NIR Fixable Viability dye (BioLegend) or To-Pro-3 (Thermo Fisher Scientific) as indicated. Evidence of apoptosis was assessed with annexin-V APC (BD Biosciences) and 7-AAD (Invitrogen) costaining according to the manufacturer’s protocol after completion of surface marker staining. Flow cytometric analysis was performed using a 5-laser Fortessa and Symphony A3 lite cytometers (Becton Dickinson). Sorting of B cells was performed using a 4-laser FACSAria II/III (Becton Dickinson). For purification of rare donor-derived populations before RNA-Seq analysis, sorting was performed twice serially to enhance purity with collection of the sorted cells in TRIzol. Analysis was performed using FlowJo software (Becton Dickinson).

### B cell purification and adoptive transfer.

Spleens of B6-CD45.1 congenic mice were processed as described above and labeled with CTV or eFluor450 (Invitrogen) proliferation dyes following the manufacturer’s instructions. Highly purified (>99%) FoB cells (CD19^+^CD93^–^CD23^hi^CD1d^lo^) were sorted using a 4-laser FACSAria II/III (Becton Dickinson). Cells were washed, resuspended in sterile PBS, and i.v. injected retro-orbitally into recipient mice (1 × 10^6^ to 1.5 × 10^6^ cells/recipient). In selected experiments, B6-CD45.2 FoB cells were transplanted instead into B6-CD45.1 or *Rag2^–/–^* recipients.

### In vivo inhibition of Notch ligands and receptors.

Mice were treated with mouse IgG2a blocking mAbs specific for Notch2 receptors (anti-NRR2) or an isotype control antibody specific to Ragweed (Genentech). Alternatively, mice received humanized IgG1 mAbs specific for Dll1 or Dll4 Notch ligands versus a mAb specific to herpes simplex virus gD glycoprotein as an isotype control (Genentech). In all cases, antibodies were administered i.p. (5 mg/kg) at days 0, 3, and 6 after B cell transfer (36, 37, 41, 58). The efficacy and specificity of each antibody batch was tested in vivo by assessing loss of Dll4-dependent T cell progenitors or Dll1-dependent MZB cells in mice (37, 59).

### Immunofluorescence imaging.

Spleens were collected from recipient mice at different time points after B cell transfer, and 5 mm fragments were fixed for 4 hours in PBS with 4% paraformaldehyde and then overnight in PBS with 30% sucrose. Tissues were cryopreserved in Tissue-Plus OCT compound (Thermo Fisher Scientific), and 10 μm sections were obtained using a cryostat. Tissue sections were hydrated and then blocked for 30 minutes at room temperature in staining solution (5% donkey serum in PBS-Tween 20) plus FcR block (clone 93; BioLegend). Antibody staining was performed overnight at 4°C in staining solution with 1–2 ng/μL of the following anti-mouse antibodies: anti-CD45.1-AF488 (clone A20; BioLegend), CD169-AF647 (clone 3D6.112; BioLegend), and laminin 1+2 (polyclonal rabbit anti-mouse, ab7463; Abcam) labeled with CF568-Mix-n-Stain antibody labeling kit (Biotium). Slides were washed with PBS, mounted using ProLong Diamond Antifade Mountant (Invitrogen), and imaged using 20× magnification lenses in a Zeiss LSM710 confocal microscope. Images were processed and analyzed using Volocity 6.3. software (PerkinElmer).

### Cell culture and ex vivo stimulation.

Sort-purified CD45.1^+^ donor-derived CD19^+^ AA4.1^–^ B cells were pelleted in complete media (RPMI, 10% FBS, 10 mM HEPES, 1× penicillin/streptomycin, 1× MEM nonessential amino acids, 100 μM Na-pyruvate, 55 μM 2β-mercapto-ethanol), and the residual volume (~100 μL) was plated in 96-well culture plates at a final volume of 200 μL/well. Cultures were supplemented as indicated with CpG DNA (0.1 μM; IDT). Cultures were incubated at 37°C in 5% CO_2_ for 48 hours.

### ELISpot assay.

ELISpot assay plates (MilliporeSigma, MSIPN4W50) were coated with capture goat anti–Ig-heavy/Ig-light antibody (Southern Biotech, 1010-01) at 2 μg/mL (1:500) in sodium carbonate/bicarbonate buffer (pH 9.6) and blocked in complete growth media. Cells were either resuspended after sorting and serially diluted across the plate (CD45.1^+^, CD19^–^) or sorted directly into complete growth media at indicated numbers of live (To-Pro-3^–^) cells/well (CpG-stimulated CD45.1^+^, CD19^+^) and incubated overnight. Biotinylated goat anti Igκ + Igλ (Southern Biotech, 1050-08, 1060-08) capture antibody was used at 0.1 μg/mL. ExtrAvidin alkaline phosphatase (Sigma-Aldrich) was used at 1:10,000 final dilution. Spots were developed using BCIP/NBT liquid substrate (Sigma-Aldrich) and 1 M sodium phosphate stop solution. Image capture, counting, and quality control were performed using the CTL Immunospot analyzer and software (Cellular Technology Limited).

### ELISA.

Purified FoB cells were isolated from C57BL/6-C20-CD45.1 mice congenic for Igh^a^ and adoptively transferred into B6 or *Rag2^–/–^* mice. Recipients were bled at days 0, 7, 14, 21, and 30 and serum was isolated by centrifugation. Nunc Maxisorb 96-well flat-bottom plates (Thermo Fisher Scientific) were coated with 10 μg/mL F(ab’)_2_ fragment goat anti-mouse IgM in sodium bicarbonate buffer and blocked with 2% BSA/PBS. Serum was serially diluted across the plate and incubated overnight at 4°C. For detection, biotin anti-mouse IgM^a^ (Igh-6^a^, BD Pharmingen) and biotin anti-mouse IgM^b^ (AF6-78, BioLegend) were diluted in blocking buffer and added at 1:3000 for 2 hours at room temperature followed by streptavidin-HRP (BD Pharmingen) at 1:10,000 for 1 hour at room temperature. Wells were developed with 3,3’,5,5’-Tetramethylbenzidine substrate solution and stopped with phosphoric acid. Plates were read on an ELISA plate reader (Molecular Devices). Concentrations were extrapolated from a standard curve generated based off the absorbance of normal mouse serum from a C57BL/6-C20-CD45.1 donor.

### RNA-Seq.

Cell populations were twice sorted directly into TRIzol (Thermo Fisher Scientific) with 0.5% 2β-mercapto-ethanol and held at –80°C until RNA preparation. RNA was prepared by TRIzol RNA extraction protocols according to the manufacturer’s instructions (Thermo Fisher Scientific). RNA was coprecipitated using glycogen as a carrier. RNA was quantified using Qubit RNA high-sensitivity fluorometric assay (Thermo Fisher Scientific). cDNA was prepared using a Clontech SMART-Seq HT RNA Kit (Takara Bio USA) according to the manufacturer’s protocol using up to 500 ng RNA as input. cDNA was quantified and qualified using HSDNA assay on an Agilent 2200 TapeStation. RNA-Seq libraries were constructed using the Illumina Nextera XT kit with 125 ng of cDNA input. Libraries were quality controlled and quantified by TapeStation and pooled at equal molar ratio prior to sequencing on Illumina HiSeq (50 bp SE v4 high output) and Illumina NextSeq500 (75bp SE v2) machines. RNA-Seq data are available in NCBI’s Gene Expression Omnibus (GEO) under the accession number GSE200219.

### Pseudoalignment and gene expression.

Transcript abundance was computed by pseudoalignment with Kallisto (60). Transcripts per million (TPM) values were then normalized and fitted to a linear model by empirical Bayes method with the voom and limma R packages (61, 62). Differential gene expression was defined as a Benjamini and Hochberg corrected *P* value of less than 0.05 and fold change greater than 2 unless otherwise noted. All data analyses and display were generated in the R statistical environment.

### Statistics.

GraphPad Prism version 9 was used to calculate statistical significance using either 1- or 2-way ANOVA or 2-tailed Student’s *t* test, as indicated in each figure. RNA-Seq analysis was performed in the R environment as described above. *P* values of less than 0.05 were considered significant.

### Study approval.

All experiments were conducted per protocols approved by the University of Michigan’s Committee on Use and Care of Animals and the University of Pennsylvania’s Office of Regulatory Affairs.

## Author contributions

DGA, BTG, JL, and BS designed research studies, performed experiments, and analyzed data. SK, EP, and AA performed experiments and analyzed data. UK, FR, BL, CWS, RJHR, TFR, JKB, and WSP provided critical reagents and/or advice. DA and IM selected the research questions, defined the overall experimental approach, and supervised the work. DGA, BTG, JL, SK, EP, DA, and IM wrote the manuscript, and all authors reviewed it. DGA, BTG, and JL share co–first authorship because of their sizable contributions to different aspects of the work, with the author order decided on the basis of the length of their involvement in the project and on DGA’s role in coordinating the work of the experimental team.

## Supplementary Material

Supplemental data

## Version 1 05/17/2022

In-Press Preview

## Version 2 07/01/2022

Electronic publication
