# Supplementary material for: Stromal Notch ligands foster lymphopenia-driven functional plasticity and homeostatic proliferation of naive B cells
Source: J Clin Invest. 2022 Jul 1;132(13):e158885. doi: 10.1172/JCI158885 (PMC9246379; doi:10.1172/JCI158885)
Supplement: Supplemental data [file jci-132-158885-s031.pdf]

Supplementary Figures

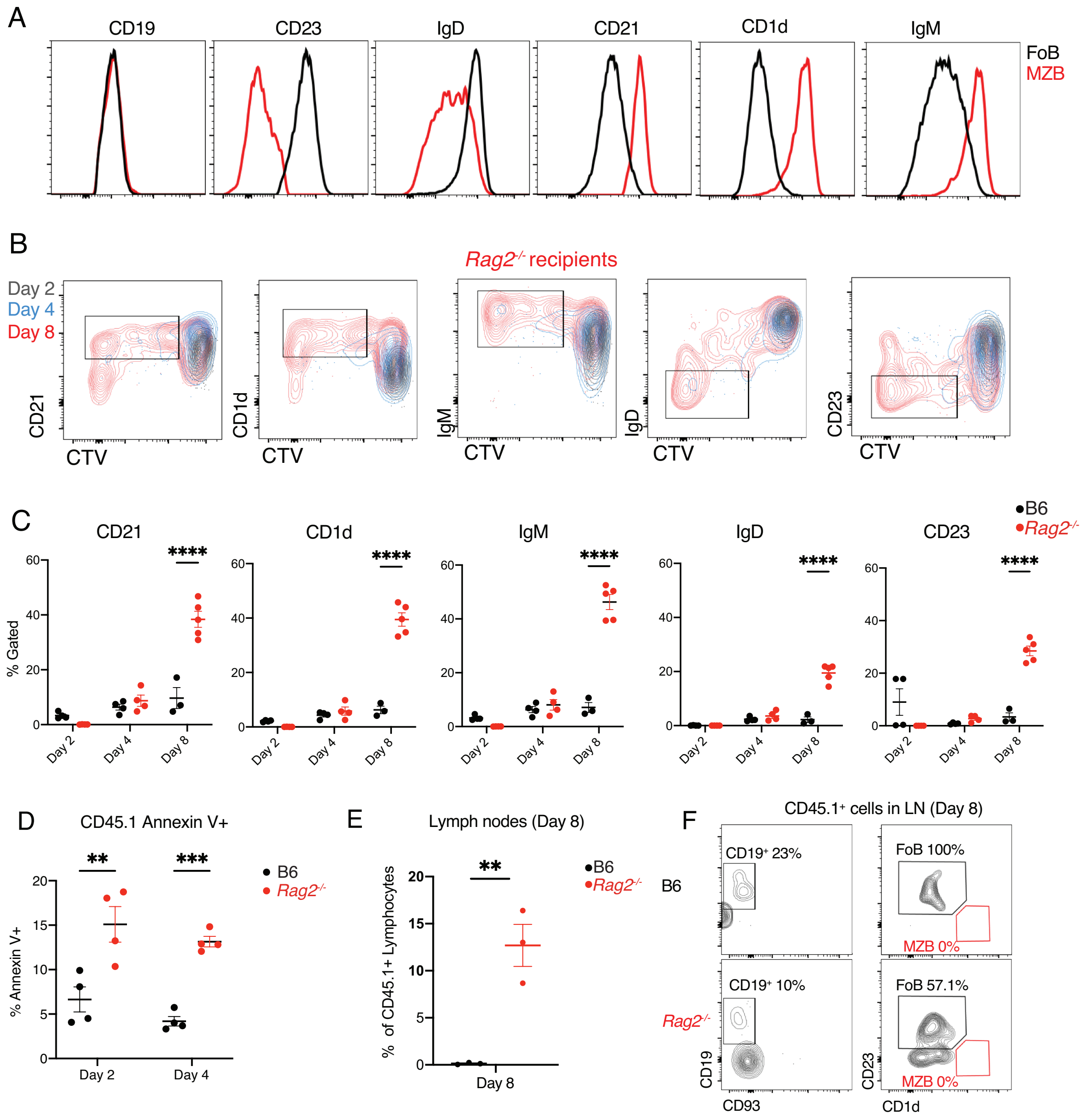

Figure S1, related to Figure 1 and 2. The acquisition of a marginal zone B-like phenotype precedes proliferation and is exclusive to the spleen. (A) Histograms depicting cell surface expression of markers indicated above in resting follicular (FoB, black) and marginal zone B cells (MZB, red); **(B)** Cell surface phenotype change in 2, 4, or 8 days (black, grey, and red respectively) following adoptive transfer with respect to cell trace violet (CTV) dilution in *Rag2*$^{-/-}$ recipients. One representative of 4 is shown; **(C)** Percentage of divided cells positive for the indicated cell surface marker on day 2, 4, or 8 in B6 (black) and *Rag2*$^{-/-}$ (red) recipients. Error bars depict SEM; **(D)** Apoptosis was measured at 2 and 4 days post adoptive transfer of FoB into B6 (black) and *Rag2*$^{-/-}$ (red) recipients. %Annexin-V^{+} fraction (including both 7-AAD^{-} and 7-

AAD$^+$) is shown as a measure of total apoptosis. **(E)** CD45.1$^+$ B cells detected in lymph nodes on day 8 after adoptive transfer of FoB cells into B6 and *Rag2*$^{-/-}$ recipients; **(F)** Percentage of CD45.1$^+$ lymphocytes detected in lymph nodes of B6 (black) or *Rag2*$^{-/-}$ recipients (red) on day 8. (C,E) **p<0.01 ***p<0.001 ****p<0.0001: two-way ANOVA **(C,D)**, Student's t test **(E)**. Each data point represents an individual mouse.

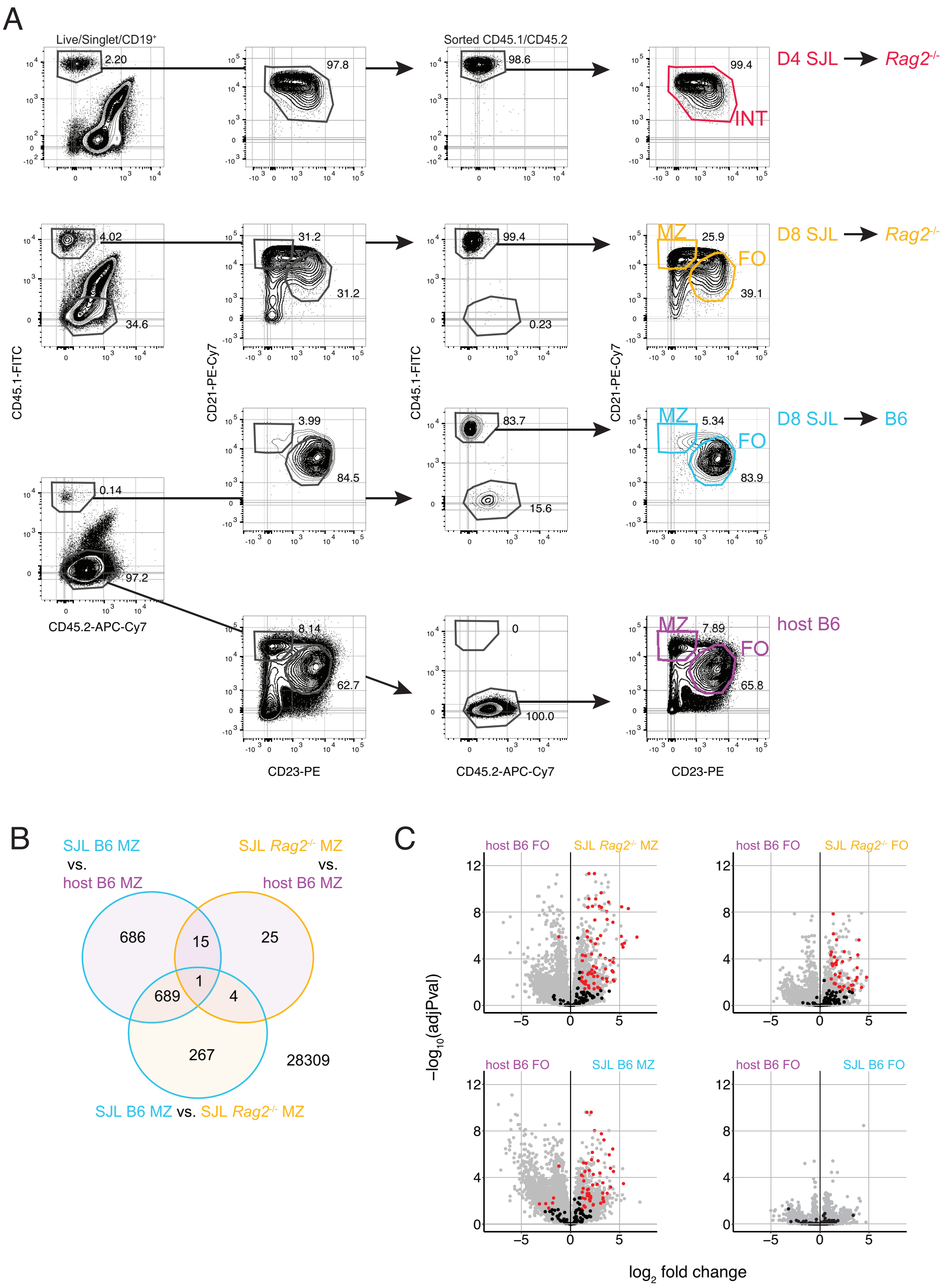

Figure S2, related to Figure 4. Follicular B cells adoptively transferred into a lymphopenic environment attain a full marginal zone B cell transcriptome. (**A**) Representative sort purification gates are shown for all samples in Figure 4. Samples were initially sorted based on CD45.1/2 expression (first column). A flow cytometry plot derived from this gate is shown for reference only in column 2. A second sort was then performed on purified CD45.1^{+} and CD45.2^{+} samples with sorting based on CD23/CD21 expression into intermediate (day 4 only) as well as MZB and FoB cell gates (day 8), as shown. CD45.2^{+} host B cells at day 8 were purified as

equivalents to normal MZB and FoB cells; (**B**) Venn diagrams displaying differential gene testing results of the indicated comparisons. Shown are the number of genes differential (adj-P value <0.01, log2 fold change >2) without respect to direction; **(C)** Volcano plots indicating the magnitude and significance of gene expression changes between indicated groups for all (grey), and highlighted (black/red) empirically defined MZB cell Notch2-regulated genes (genes significantly downregulated in MZB cells after 24 hours anti-Notch2 antibody blockade) (Gaudette et al., *J Clin Invest* 131(20), e151975, 2021). Red color indicates significance (adj-P < .05; log2 fold change >1).

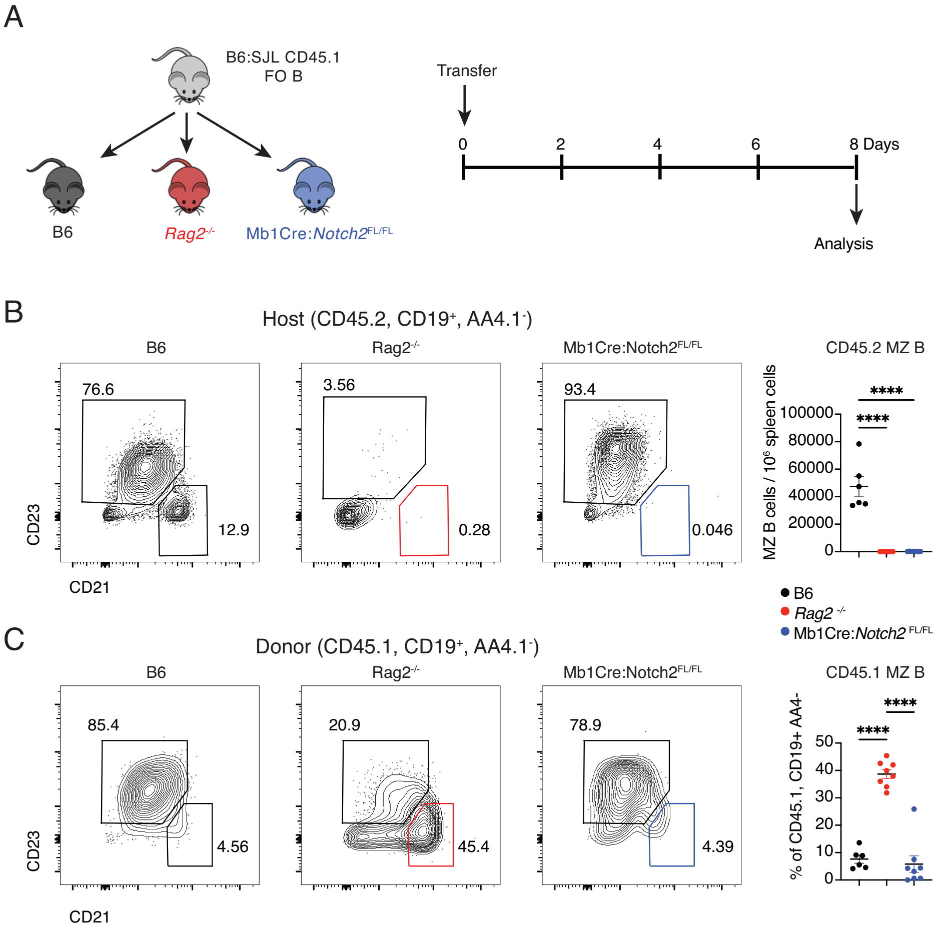

Figure S3. Lack of host marginal zone B cells alone is not sufficient to drive the transdifferentiation of adoptively transferred follicular B cells into MZ-like B cells. Congenically marked FoB cells were labeled with CellTrace Violet and adoptively transferred into either B6, *Rag2*$^{-/-}$, or *Mb1-Cre;Notch2*$^{f/f}$ recipients, which selectively lack MZB cells. Flow cytometric analysis of splenocytes performed 8 days post transfer. **(A)** Schematic depiction of the experimental design. **(B)** Representative FoB vs. MZB cell gating is shown for the host spleen B cell compartment in each genotype (gated on live, singlet, CD45.2^{+} CD19^{+} AA4.1^{-}) and MZB cells are quantified per spleen (right) for all animals. **(C)** Representative FoB and MZB cell gating is shown for the donor B cell compartment (gated on live, singlet, CD45.1^{+} CD19^{+} AA4.1^{-}) in each genotype. The MZB cell phenoype as a percentage of donor-derived B cells is shown on the right. **(B,C)** Pooled results from two independent experiments are shown as individual animal data points (n=6-8 per group, mean $\pm$ SEM). ****p<.0001 one way ANOVA, Tukey's correction for multiple comparisons.
